# Context and Perceptual Salience Influence the Formation of Novel Stereotypes via Cumulative Cultural Evolution

**DOI:** 10.1111/cogs.12560

**Published:** 2017-11-02

**Authors:** Jacqui Hutchison, Sheila J. Cunningham, Gillian Slessor, James Urquhart, Kenny Smith, Douglas Martin

**Affiliations:** ^1^ Division of Psychology University of Abertay; ^2^ School of Psychology University of Aberdeen; ^3^ School of Philosophy, Psychology and Language Sciences University of Edinburgh

**Keywords:** Stereotypes, Stereotype formation, Cultural evolution, Social cognition, Person perception

## Abstract

We use a transmission chain method to establish how context and category salience influence the formation of novel stereotypes through cumulative cultural evolution. We created novel alien targets by combining features from three category dimensions—color, movement, and shape—thereby creating social targets that were individually unique but that also shared category membership with other aliens (e.g., two aliens might be the same color and shape but move differently). At the start of the transmission chains each alien was randomly assigned attributes that described it (e.g., *arrogant*,* caring*,* confident*). Participants were given training on the alien‐attribute assignments and were then tested on their memory for these. The alien‐attribute assignments participants produced during test were used as the training materials for the next participant in the transmission chain. As information was repeatedly transmitted an increasingly simplified, learnable stereotype‐like structure emerged for targets who shared the same color, such that by the end of the chains targets who shared the same color were more likely to share the same attributes (a reanalysis of data from Martin et al., 2014 which we term Experiment 1). The apparent bias toward the formation of novel stereotypes around the color category dimension was also found for objects (Experiment 2). However, when the category dimension of color was made less salient, it no longer dominated the formation of novel stereotypes (Experiment 3). The current findings suggest that context and category salience influence category dimension salience, which in turn influences the cumulative cultural evolution of information.

## 1 Introduction

Information changes incrementally as it is repeatedly passed from person to person, a phenomenon often termed “cultural evolution.” Recent research suggests that the cumulative process of cultural evolution could help explain fundamental aspects of human culture such as the origins of social stereotypes (Martin et al., 2014) and the evolution of language (Kirby, Cornish, & Smith, 2008). Using laboratory‐based experiments to examine how information changes as it passes from person to person, it is not only possible to gain a greater understanding of how knowledge, such as societal stereotypes, evolves but also to illuminate the underlying biases that drive the process of cultural evolution. The current research examines whether the context in which people encounter information and the relative salience of category dimensions influences the cumulative cultural evolution of novel stereotype‐like structure.

Stereotypes are an example of a highly structured, simplified, and easy‐to‐learn information system (Brewer, 1988; Fiske & Neuberg, 1990), whereby membership of social groups is associated with the possession of certain attributes (e.g., scientists are geeky, women like the color pink, Scottish people wear kilts; Allport, 1954). Martin et al. (2014) recently showed that novel stereotypes can form spontaneously when information is repeatedly passed from person to person down transmission chains. At the start of each chain the experimenters randomly assigned personality attributes to “alien beings” that were individually unique but that also shared category membership (i.e., some aliens were the same shape, some were the same color, and some moved in the same way). The first participant in a chain (Generation 1) attempted to learn which attributes were associated with each alien; whatever this person recalled was used as the basis of the training materials for the next person in the chain (i.e., Generation 2). The process of using the test responses from one person as the training materials for the next was repeated (as in the children's game often called “Chinese Whispers” or “Telephone”) seven times per chain to create social transmission chains of seven generations.

Martin et al. (2014) found that an initially random set of information that was difficult to remember became increasingly learnable as it passed through the generations. Any tendency toward structure evidenced in the attribute assignments of one participant was detected and amplified in the recollections of the next. Over multiple generations a stereotype developed, with category features (i.e., color, shape, movement) becoming so strongly associated with the possession of specific attributes that they could be used to accurately infer information about social targets that had never been seen before (e.g., by the end of one chain all green aliens were agreed to be “arrogant” and “pushy,” while red aliens were thought to be “shy”). As social information was repeatedly communicated, it evolved to become an easily learnable categorically structured stereotype knowledge system.

One unanticipated pattern in the data from Martin et al. (2014) was the emergence of a single category dimension—color—that appeared to dominate the way that stereotype structure evolved. By the final generation, more than 70% of the transmission chains had a structure dominated by color (i.e., aliens who shared the same color had more shared attributes than those that matched on shape or movement). These data were not statistically analyzed by Martin et al., but this pattern suggests people were more likely to use the category dimension of color as an organizing principle than they were either shape or movement and that this tendency influenced the stereotypes that formed via cultural evolution.

While color is often deemed relatively unimportant when people are categorizing unfamiliar objects (Baldwin, 1989; Graham & Poulin‐Dubois, 1999), it is of central importance when people are categorizing unfamiliar people (Mason, Cloutier, & Macrae, 2006). People's impressions of unfamiliar others often begin with the identification of the social categories to which they perceive a person belongs (e.g., sex, age, race; Brewer, 1988; Fiske & Neuberg, 1990). Research stresses the importance of both color and tone as category‐specifying cues for identifying both age (i.e., hair color/quantity and skin tone—Berry & McArthur, 1986; Burt & Perrett, 1995; Mark et al., 1980; for a review, see Enlow, 1982) and race (i.e., skin tone and the shape of individual face features—Enlow, 1982; Levin, 2000; MacLin & Malpass, 2001; for a review, see Maddox, 2004). Indeed, such is the importance of color for the identification of race that reducing the salience of this feature significantly impedes people's ability to make categorical race judgments (Cloutier & Macrae, 2007).

The idea that color might be a dominant cue when categorizing social targets is consistent with established stereotype activation findings showing that color cues associated with ethnicity often dominate person perception (Dunham, Dotsch, Clark, & Stepanova, 2016). Even if a distinction based on a person's ethnicity is irrelevant to the task at hand, evidence suggests that people are perceptually sensitive to this category cue, extracting the information from faces in a seemingly automatic manner (Freeman, Nakayama, & Ambady, 2013; Martin et al., 2015). However, while skin tone and race might dominate social categorization under some circumstances there is also abundant evidence that such effects are dependent on the context in which social targets are encountered (e.g., Blair, 2002; Casper, Rothermund, & Wentura, 2010; Gilbert & Hixon, 1991; Mitchell, Nosek, & Banaji, 2003). For example, Mitchell et al. (2003) demonstrated that when highly regarded Black athletes were categorized as a function of their occupation, they elicited positive attitudes, but when their race was made salient, the elicited attitude was qualitatively different. This suggests that some category dimensions, such as color, might be more likely than others to impact how stereotypes form through cumulative cultural evolution but that the dominance of such category dimensions is likely to be context dependent.

The suggestion that the influence exerted on the cumulative cultural evolution of information by specific category dimensions might be context dependent is also consistent with the finding that color has little influence on the formation and evolution of novel languages (Kirby et al., 2008). The method and materials Martin et al. used to explore stereotype formation were adapted from those used by Kirby et al. (2008) to examine the effects of cultural transmission on the evolution of artificial languages: Kirby et al. used “alien objects” paired with letter strings, whereas Martin et al. used “alien beings” paired with personality attributes. While there were strong parallels between the development of novel languages in Kirby et al. (2008) and the development of novel stereotypes in Martin et al. (2014)—both were characterized by reduced complexity, increased structure, and increased learnability—there was also evidence that their structural evolution was quite different. Importantly, there was no evidence of bias toward the color category dimension in the development of Kirby et al.'s artificial languages. Indeed, as the novel languages passed down the chains, they became underspecified on the color category dimension, such that shape and movement were more likely to be encoded in the language than color. A recent reanalysis of the data from Kirby et al. (2008) concluded that movement was the dominant category dimension; in a subsequent experiment where this category dimension was absent (i.e., objects differed in shape, color, and number), the evolving novel languages were likely to be structured around the shape category dimension (Beckner, Pierrehumbert, & Hay, 2017).

Alternatively, it may be that the dominance of color in the novel stereotypes from Martin et al. (2014) is simply due to salience, rather than a bias to organize social stereotypes around skin color. Perceptual saliency plays an important role in what cues individuals attend to in the environment (Macrae & Bodenhausen, 2000), and there is abundant evidence that social category cues differ in their relative perceptual salience (e.g., Cloutier, Mason, & Macrae, 2005; Cloutier & Macrae, 2007; Macrae & Martin, 2007; Martin & Macrae, 2007; for a review, see Martin & Macrae, 2010). For example, the most pervasive social categories in society are ones that are easily perceived such as sex, age, and race (Brewer, 1988; Fiske, 1993; Fiske & Neuberg, 1990). However, people can belong to multiple categories at any one time and increasing or reducing the saliency of a category dimension can influence what stereotype is cued and thus influence the perceiver's subsequent cognition and behavior (Mitchell et al., 2003). Therefore, it is possible that perceptual saliency plays a role not only in stereotype activation but in novel stereotype formation, with perceptually salient category cues dominating the stereotype formation process. If this is the case, then reducing the perceptual saliency of the color category dimension should result in that category no longer dominating the emergent stereotype. This prediction is consistent with findings in the language evolution literature showing that less salient category dimensions are less likely to be encoded in evolving languages (Silvey, Kirby, & Smith, 2015).

In order to distinguish between these two possible explanations for the dominance of color in the evolving stereotypes of Martin et al. (2014)—color is preferred in the context of social category learning, or color is preferred when it is salient—in this paper, we examined how context and perceptual salience influence the evolution of stereotypes in the laboratory. Specifically, we re‐examined the data from Martin et al. (2014) to establish whether there is statistically robust evidence that the formation of novel social category stereotypes is dominated by the category dimension of color (Investigation—a reanalysis of data from Martin et al., 2014). We then conducted two further experiments with new samples to investigate whether the formation of novel object category stereotypes is dominated by the category dimension of color (Experiment 2) and if reducing the perceived perceptual difference between color categories attenuates the bias toward dominance of the color category dimension and thereby influences the development of novel stereotypes (Experiment 3).

## 2 Investigation—A reanalysis of data from Martin et al. (2014)

The aim of the investigation was to reanalyze previously published data^1^ to establish whether the impression based on the data that the formation of novel social stereotypes is dominated by the category dimension of color is verified statistically. For expository purposes, we will refer to this investigation as Experiment 1 hereafter. In addition to establishing whether people share a bias to categorize novel social targets based on color, we also examined whether such effects might be driven by people imposing structure in a top‐down manner based on pre‐existing semantic associations between the individual color features and specific attributes (e.g., people might share a bias toward associating the color *red* with the attribute *warm*). If any higher levels of color structure are driven by a shared bias to associate the color of aliens with semantically related attributes, we would expect the frequency with which those attributes appeared with each color to differ from that expected by chance.

### 2.1 Method

The method is as is reported in Martin et al. (2014); since all three experiments here use the same method, we describe the method in full.

#### 2.1.1 Participants

The sample reported here is a subset of the sample from Martin et al. (2014). Eighty‐four undergraduate students from the University of Aberdeen (58 females) took part in Experiment 1; participants either received course credit or were reimbursed for their time. On arriving in the laboratory, participants were informed that they would be taking part in an experiment examining how we form impressions of other people and that their task would be to try and remember the personality attributes associated with novel “alien” social targets. Participants were then assigned to the next sequential generation in an active chain before being given full instructions about the nature of the tasks they would be completing. Importantly, participants were not informed about the social transmission aspect of the experiment until they were debriefed at the end of the testing session.

#### 2.1.2 Stimulus materials

**Figure 1:**
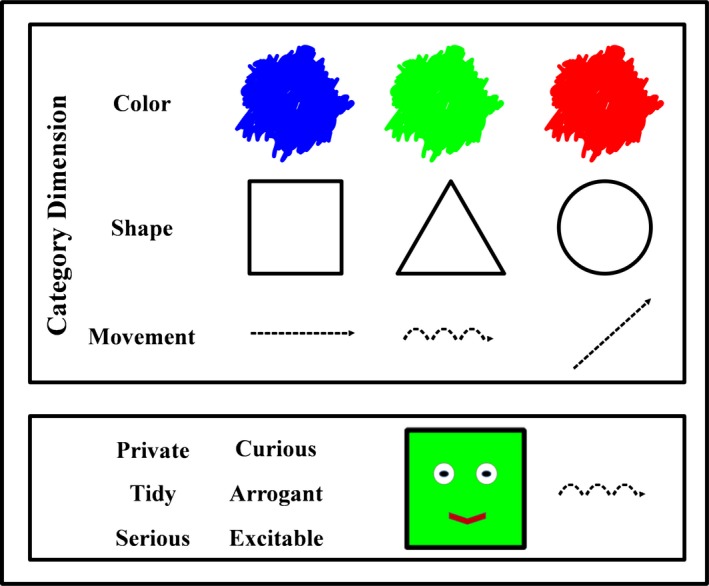
Alien category dimensions and features (top panel); example of an alien stimulus with associated attributes (bottom panel). This figure was originally printed in Martin et al. (2014).

*Alien images*: Each alien was represented by a simple line drawing combining *features* from the category dimensions of shape (*circle, square, triangle*), color (*blue, green, red*)*,* and movement (*bouncing, diagonally, horizontally*; see Fig. 1). Factorial combination of the three features from each category dimension resulted in 27 unique aliens, each of which shared some category features with some other aliens. For example, a pair of aliens might share one category feature such as color, but some alien pairs might share two category features such as color *and* shape. No alien pair shared all three features, thus ensuring each alien was unique. Each alien image was around 12° × 6° visual angle as viewed from an approximate distance of 57 cm.

Attributes: The attributes used to describe each alien were drawn from a total pool of 48 attributes that could be used to describe a human (e.g., *arrogant*,* caring*,* confident:* for a list of attributes, see *Supplementary Materials* S1). The attributes had been screened previously using a separate sample of equivalent undergraduates to ensure they were likely to be unambiguously familiar to the participants in the transmission chain experiment. Six attributes were used to describe each alien.

#### 2.1.3 Procedure

**Figure 2:**
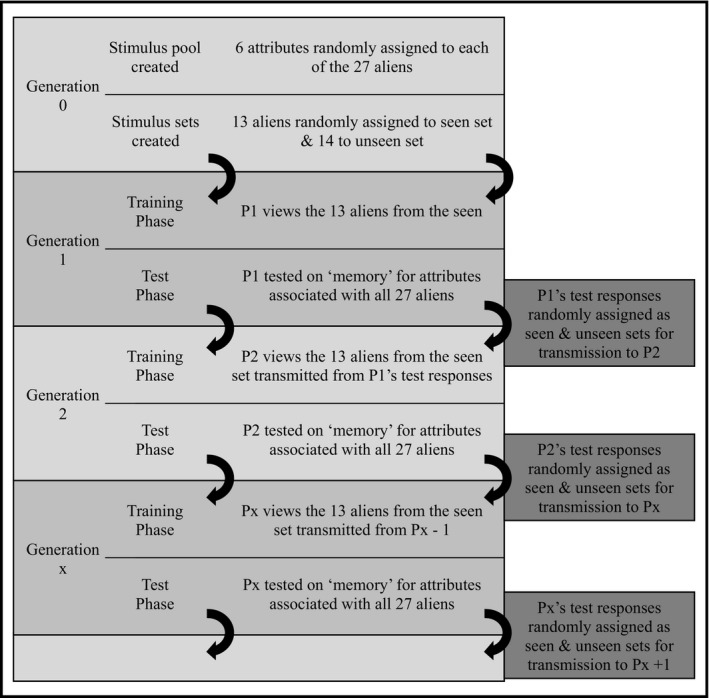
Outline of the processes of social transmission of information across multiple generations (adapted from Martin et al., 2014).

*Overview (see Fig.* 2 *):* Before the first participant in a chain came in to the laboratory for testing, six personality attributes were randomly assigned to each alien to create the Generation 0 alien/attribute pool. During an initial training phase, the first participant in a chain (Generation 1) was shown a randomly selected subset of 13 of the 27 aliens from this pool and attempted to learn their associated attributes. During the subsequent test phase, participants were shown all 27 aliens—both the 13 aliens they had encountered during training and the 14 aliens that had remained unseen during training—and were asked to select the six attributes associated with each alien. The test responses to all 27 aliens from Generation 1—whether correct or incorrect—acted as the pool of materials from which the next participant's training materials were created. This involved randomly dividing the aliens and their associated attributes (i.e., the attributes that had been assigned at test by the participant at the previous generation) into new seen and unseen sets; the 13 aliens in the newly created seen set were then used as the training materials for Generation 2 with the remaining 14 aliens being withheld. The process of using a random sample of the test responses from one generation as the training materials for the next was repeated seven times per chain.

### 2.2 Dependent variables

Each chain was effectively treated as a single “participant,” while each generation within a chain (i.e., individual participant) was treated as a level of a repeated measures factor.

#### 2.2.1 Total attributes

The total attributes dependent measure was merely the total number of unique attributes used across the entire pool of aliens at a given generation (i.e., the number of unique attributes assigned randomly at Generation 0 or assigned by participants at test). The total attributes score can vary between a minimum of 6 and a maximum of 48. For example, if a participant used the same 6 attributes to describe all 27 aliens, then the total attributes score would be 6; however, if across all of the aliens, a participant made use of all 48 attributes, then the total attributes score would be 48.

#### 2.2.2 Accuracy

Accuracy was the percentage of attribute/alien association responses made during the test phase that correctly matched the attribute/alien associations *of the previous generation*. Accuracy at Generation 1 was the percentage of attribute/alien test responses that matched the random attribute/alien pool allocated at Generation 0; similarly, accuracy at Generation 2 was the percentage of attribute/alien test responses that matched the test responses at Generation 1 and so on. Importantly, accuracy refers both to items that had been seen during training (i.e., do the participant's responses match those of the aliens they were trained on?) and the items that were unseen (i.e., do the participant's responses match the responses of the previous generation for unseen aliens, even though these pairings of aliens and attributes were not seen during training?).

#### 2.2.3 Structure

To quantify the amount of structure at each generation of a chain, we first calculated the overlap in attributes between each individual alien and the other 26 aliens in the same generational pool; this gave us 351 within‐generation alien overlap scores. Any two aliens that had no overlap in attributes were given a score of 0, any two aliens that shared one attribute were given a score of 1, and so on up to a maximum score of 6 for aliens that had six identical attributes. Based on the overlap scores between all aliens, we then calculated three *raw structure scores* by taking the mean across all pairs of aliens who shared a single category dimension feature. This meant we had separate structure scores for those aliens who were the same color but differed in shape and/or movement, for those aliens who were the same shape but differed in color and/or movement, and for those aliens who moved in the same way but who were a different color and/or shape.

Rather than using the raw structure score as a dependent measure in its own right, we converted the raw scores to standard scores; raw structure scores typically increase by chance as the total number of unique attributes used decreases (e.g., if only eight unique attributes were randomly assigned across the 27 aliens, one would expect very high structure scores because there would be considerable chance overlap between aliens, whereas if 48 attributes were randomly assigned across the 27 aliens, one would expect much lower structure scores because there would be considerably less chance overlap between aliens). To examine whether the raw structure score in a generation was greater than would be expected by chance, we generated random simulated structure data by running Monte Carlo simulations (12,000 runs) based on a random allocation of attributes to aliens, limited to the total attributes used at each generation. The simulated structure data were then used as a comparison dataset in order to calculate *z*‐scores for the raw structure data—these *z*‐scores are our dependent measure of structure.

### 2.3 Results and discussion

While the main aim of the reanalysis of the subset of data from Martin et al. (2014) was to examine whether stereotypes were more likely to form around the category dimension of color, we began our analyses by running paired samples *t* tests to determine whether the basic pattern of effects from the subset of data used here (i.e., 12 chains) matched those previously reported for the entire dataset (i.e., 24 chains).^2^ Specifically, we ran paired samples *t* tests to examine whether there was a significant reduction in the number of total attributes used between Generation 1 and Generation 7, and a significant increase in accuracy at Generation 7 relative to Generation 1 as was found in Martin et al. (2014). Paired *t* tests showed that the total number of attributes used to describe all 27 aliens at Generation 7 (*M* = 24) was lower than at Generation 1 (*M *=* *39; *t*(11) = 6.53, *p *<* *.001).

While there was some correspondence between attributes assigned at Generation 0 and those assigned at Generation 7 (*M *=* *13.1% of attributes preserved to Generation 7), one‐sample *t* tests revealed this was not greater than would be expected by chance (chance = 12.4%) even for aliens whose attributes were seen during the training phase by the Generation 1 participant [*M* = 14.1%; *t*(11) = 1.82, *p *=* *.095]; unsurprisingly, attributes assigned at Generation 0 but unseen by the participant at Generation 1 were no more likely than chance to be retained at the end of the chain [*M* = 12.0%; *t*(11) = −0.296, *p *=* *.773].

Paired *t* tests also revealed greater accuracy for seen aliens at Generation 7 (*M *=* *47%) relative to Generation 1 (*M *=* *19%; *t*(11) = 5.72, *p *<* *.001) and unseen aliens at Generation 7 (*M *=* *41%) relative to Generation 1 (*M *=* *13%; *t*(11) = 5.05, *p *<* *.001). These results suggest that the pattern of data in the subset of 12 chains to be reanalyzed for effect of color here matches the pattern from the larger dataset of Martin et al. (2014). We then proceeded to examine the effects of theoretical interest—whether there is evidence that the color category dimension exerted greater influence on the formation and evolution of novel stereotypes than did the other category dimensions.

#### 2.3.1 Structure by category dimension

**Figure 3:**
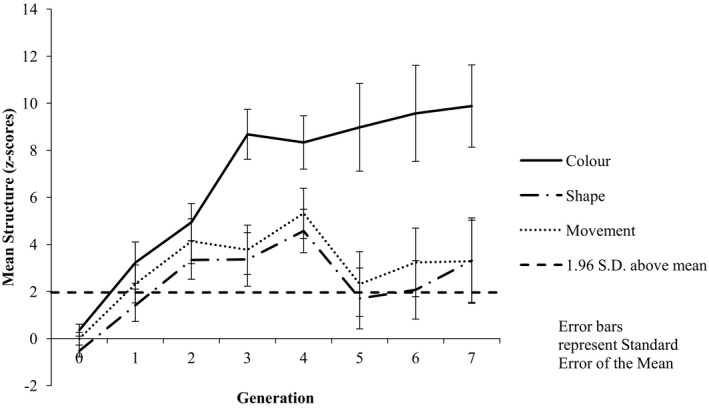
Mean level of structure at each generation by category feature. Increasing positive *z*‐scores indicate increasing structure, with *z*‐scores greater than +1.96 indicating higher structure than would be expected by chance (α = 0.05).

Inspection of the mean structure associated with each category dimension across the generations suggests a bias toward color categorization, with numerically higher structure associated with color in nine chains; three chains showed numerically higher structure associated with movement, and no chains showed higher structure associated with shape (see Fig. 3 for mean structure across chains). We analyzed structure data using an 8 (Generation: G0–G7) × 3 (Shared Feature: color vs. shape vs. movement) repeated measures anova. The results revealed a main effect of Generation [*F*(7, 77) = 8.78, *p *<* *.001, $\eta_{p}^{2}$ = 0.444], with pairwise comparisons indicating significantly more structure at G7 (*M* = 5.49) than at G0 (*M* = −0.058; *p* = .001). This analysis also revealed a main effect of Shared Feature [*F*(2, 22) = 12.33, *p *<* *.001, $\eta_{p}^{2}$ = 0.528]; pairwise comparisons revealed that there was significantly more structure associated with color (*M* = 6.75) than either shape (*M* = 2.41; *p* = .003) or movement (*M* = 3.05; *p* = .007) and that there was significantly more structure associated with movement than with shape (*p* = .009). The analysis also revealed a significant Generation by Shared Feature interaction [*F*(14, 154) = 2.14, *p *=* *.012, $\eta_{p}^{2}$ = 0.163]. Examination of this interaction revealed that at Generation 0 there was no difference in the amount of structure between color (*M* = 0.358) and shape (*M* = −0.527; *p *=* *.081) or movement (*M* = −0.005; *p* = .443), or shape and movement (*p* = .071); however, at Generation 7 there was significantly more structure associated with color (*M* = 9.82) than either shape (*M* = 3.31; *p* = .047) or movement (*M* = 3.29; *p* = .039).

#### 2.3.2 Structure by color category features

**Figure 4:**
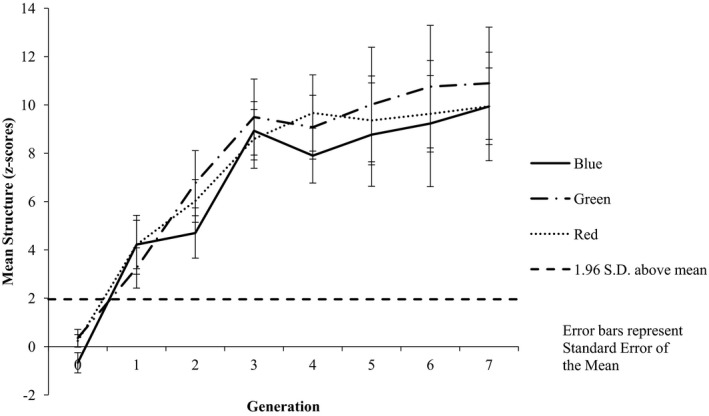
Mean level of structure at each generation by color. Increasing positive *z*‐scores indicate increasing structure, with *z*‐scores greater than +1.96 indicating higher structure than would be expected by chance (α = 0.05).

Inspection of the mean structure associated with the individual features of the color category dimension across the generations suggests no obvious bias toward particular colors (see Fig. 4). This impression is supported by an 8 (Generation: G0–G7) × 3 (Shared Feature: blue vs. green vs. red) repeated measures anova, which revealed a main effect of Generation [*F*(7, 77) = 8.71, *p *<* *.001, $\eta_{p}^{2}$ = 0.442], with pairwise comparisons indicating significantly more structure at G7 (*M* = 10.26) than at G0 (*M* = −0.027; *p* = .001); there was no evidence of a main effect of Shared Feature [*F*(2, 22) = 1.44, *p *=* *.259, $\eta_{p}^{2}$ = 0.115] nor of a significant Generation by Shared Feature interaction [*F*(14, 154) = 0.313, *p *=* *.992, $\eta_{p}^{2}$ = 0.028].

#### 2.3.3 Color/Attribute relationship frequencies

To establish whether the dominant structure associated with the color category dimension was driven by a bias toward existing semantic associations between individual attributes and individual color features, we examined the frequency with which each attribute was paired with each color feature (e.g., was the attribute “warm” paired with the semantically related color “red” more frequently than would be expected by chance; see *Supplementary Materials 1*). We used one‐sample *t* tests to compare the measured test phase frequency with which each attribute was associated with each color with the frequency that would be expected by chance. To compare the association between each of the 48 attributes and each of the three colors, we ran a total of 144 comparisons; we used the Bonferroni method to adjust the critical *p*‐value to account for the 48 multiple comparisons made within color (thus to achieve significance *p *<* *.001). Examination of these comparisons revealed that no attributes appeared with an individual color more often than would be expected by chance. However, seven attributes appeared with an individual color or colors less frequently than would be expected by chance. Of those, two attributes appeared less frequently for only one color (*imaginative* with red; *sensible* with green), three attributes appeared less frequently for two colors (*offensive* with both green and red; *reserved* with both blue and red; sensitive with both blue and red), and two attributes appeared less frequently with all three colors (*easy‐going* and *patient*). Five of these seven attributes (i.e., *patient*,* reserved*,* sensitive*,* offensive*, and *imaginative*) appeared with an individual color or colors less frequently than would be expected by chance.

The data from Experiment 1 are consistent with the category dimension of color dominating the formation of stereotypes for novel social targets in the current context. In line with previous findings there was evidence that as information passed down the chains it became increasingly structured, increasingly simplified, and, consequently, increasingly learnable (Kirby et al., 2008; Martin et al., 2014). We found that significantly more structure accrued for the color category dimension than either shape or movement. Importantly, there was no evidence that the dominance of the color category dimension was a consequence of bias toward categorizing based on a specific color (i.e., different colors dominated across different chains). Neither was there evidence that the dominance of the color category dimension was driven by bias toward existing semantic associations between individual attributes and individual color features (i.e., different color‐attribute combinations emerged across different chains).

The results observed in Experiment 1 could be a consequence of the social nature of the task or the fact that color is particularly salient for the stimuli used within the context of the experiment. Because studies examining novel language evolution (Kirby et al., 2008) typically use objects as the target stimuli of interest, it is possible that the social targets used in Experiment 1 tapped a different, more salient bias toward categorizing based on color. This is plausible given evidence that color and skin tone are important cues for real‐world social categorizations (Mason et al., 2006) such as age (Berry & McArthur, 1986; Burt & Perrett, 1995; Mark et al., 1980) and race (Enlow, 1982; Levin, 2000; MacLin & Malpass, 2001). In order to examine whether it was the context of perceiving social targets that drove the dominance of the color category dimension in Experiment 1 (i.e., remembering information about alien beings), in Experiment 2, we investigated whether the color category dimension dominates the formation of novel stereotypes when the context requires perceiving (i.e., remembering information about) novel alien objects.

## 3 Experiment 2: Categorization of objects

Given the importance of color as a category‐specifying cue to the race and age of unfamiliar people (Mason et al., 2006), it is possible that a similar sensitivity could explain the observed bias toward the color category dimension when people were remembering information about the novel social targets in Experiment 1. This suggests that people's propensity to categorize novel social targets based on the dimension of color, as seen in Experiment 1, might be specific to social targets.

The aim of Experiment 2 was to examine whether in an experimental context when people were learning information about novel alien objects, rather than novel alien beings, stereotype‐like structure would form around different category dimensions than seen in Experiment 1. Experiment 2 used an identical method to that of Experiment 1 with two exceptions; (a) target stimuli represented alien objects (Kirby et al., 2008); (b) attributes were applicable to objects. If, in contrast to Experiment 1 where participants seemed to be biased to form social categories around the dimension of color, people possess a bias toward categorizing objects according to shape (Davidoff & Ostergaard, 1988; Diesendruck & Bloom, 2003; Goldstone, 1995; Mash, 2006) or movement (e.g., Beckner et al., 2017; Kirby et al., 2008), we would expect to find greater structure associated with either shape or movement than color. If, in contrast, the dominance of color seen in Experiment 1 is simply due to the salience of color in our stimuli, we would expect to see the results from Experiment 1 replicated with the modified stimuli.

### 3.1 Method

#### 3.1.1 Participants

A new sample of 77 undergraduate participants (51 females) took part in Experiment 2. On arriving in the laboratory, participants were informed that they would be taking part in an experiment examining how we learn information about novel stimuli and that their task would be to try and remember the attributes associated with novel “alien” objects.

#### 3.1.2 Materials

**Figure 5:**
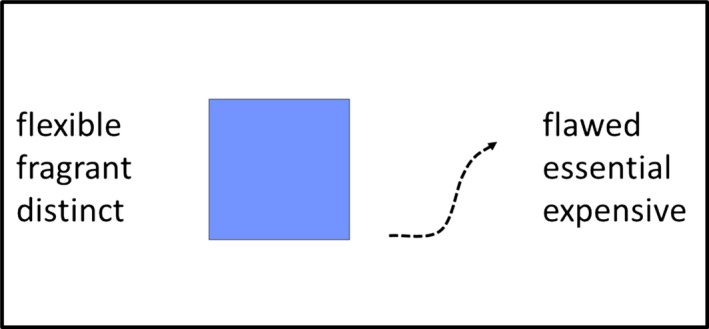
Example of alien object stimuli. The eyes and mouth were removed from the stimuli, and the attributes were replaced with adjectives appropriate to objects.

The materials were identical to those described in the Experiment 1 with the following exceptions. *Alien Images:* We manipulated the original alien images using Adobe Photoshop to remove the eyes and mouths from the stimuli (see Fig. 5). *Attributes:* The attributes used to describe each alien object were drawn from a total pool of 48 attributes that could be used to describe properties of objects (e.g., *expensive, flexible, shiny*). The attributes had been screened previously using a separate sample of equivalent undergraduates to ensure that they would be likely to be unambiguously familiar to the participants in the transmission chains. Six attributes were used to describe each alien object. The full set of attributes is listed in Supplementary Materials S1.

#### 3.1.3 Procedure

The procedure was identical to that described for Experiment 1.

#### 3.1.4 Dependent variables

These were identical to those used in Experiment 1.

### 3.2 Results and discussion

Paired *t* tests revealed that the total number of attributes used to describe all 27 aliens at Generation 7 (*M* = 18) was lower than at Generation 1 (*M *=* *38; *t*(10) = 6.21, *p *<* *.001). While there was some correspondence between attributes assigned at Generation 0 and those assigned at Generation 7 (*M* = 12.8% of attributes preserved to Generation 7), one‐sample *t* tests revealed this was not greater than would be expected by chance (chance = 12.4%) even for aliens whose attributes were seen during the training phase by the Generation 1 participant [*M* = 13.4%; *t*(10) = 1.18, *p* = .267]; attributes assigned at Generation 0 but unseen by the participant at Generation 1 were no more likely to appear at the end of the chain than expected by chance [*M* = 12.2%; *t*(10) = −0.296, *p* = .863].

Paired *t* tests also revealed greater accuracy for seen aliens at Generation 7 (*M* = 49%) relative to Generation 1 (*M* = 22%; *t*(10) = 4.98, *p *<* *.01) and unseen aliens at Generation 7 (*M* = 44%) relative to Generation 1 (*M* = 12%; *t*(10) = 6.44, *p *<* *.001). These results replicate the pattern found in Experiment 1.

#### 3.2.1 Structure

**Figure 6:**
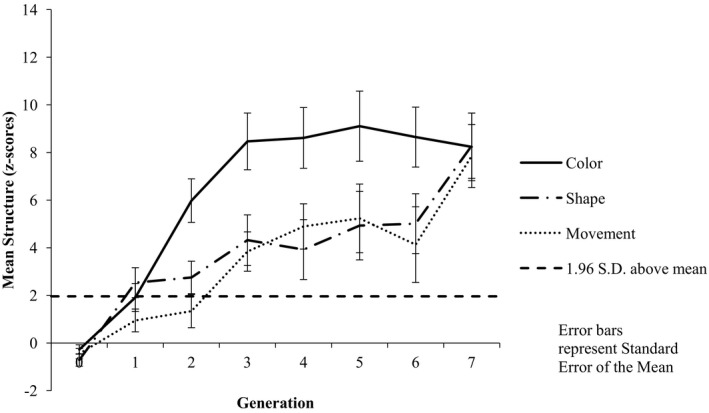
Mean level of structure at each generation by category feature. Increasing positive *z*‐scores indicate increasing structure, with *z*‐scores greater than +1.96 indicating higher structure than would be expected by chance (α = 0.05).

Inspection of the mean structure associated with each category dimension across the generations suggests a bias toward color categorization, with numerically higher structure associated with color in nine chains, relative to three chains for shape and no chains for movement (see Fig. 6). The structure data were analyzed using an 8 (Generation: G0–G7) × 3 (Shared Feature: color vs. shape vs. movement) repeated measures anova. The results revealed a main effect of Generation [*F*(7, 70) = 12.46, *p *<* *.001, $\eta_{p}^{2}$ = 0.555], with pairwise comparisons indicating significantly more structure at G7 (*M* = 8.12) than at G0 (*M* = −0.479; *p *<* *.001). The results also revealed a main effect of Shared Feature [*F*(2, 20) = 10.07, *p *=* *.001, $\eta_{p}^{2}$ = 0.502]; pairwise comparisons revealed that there was significantly more structure associated with color (*M* = 6.34) than either shape (*M* = 3.88; *p* = .018) or movement (*M* = 3.47; *p* = .003) but that there was no significant difference in the level of structure between shape and movement (*p* = .276). The analysis also revealed a significant Generation by Shared Feature interaction [*F*(14, 140) = 2.64, *p* = .002, $\eta_{p}^{2}$ = 0.209]. Examination of the interaction revealed that at G0 there was no difference in the amount of structure between color (*M* = −0.273) and either shape (*M* = −0.713; *p* = .290) or movement (*M* = −0.450; *p* = .642), or shape and movement (*p* = .320); however, at G3 there was significantly more structure associated with color (*M* = 8.47) than either shape (*M* = 4.32; *p* = .036) or movement (*M* = 3.84; *p* = .009). By G7 there was no difference in the amount of structure associated with color, shape, or movement (all *p*s > .90).

#### 3.2.2 Structure by color category features

**Figure 7:**
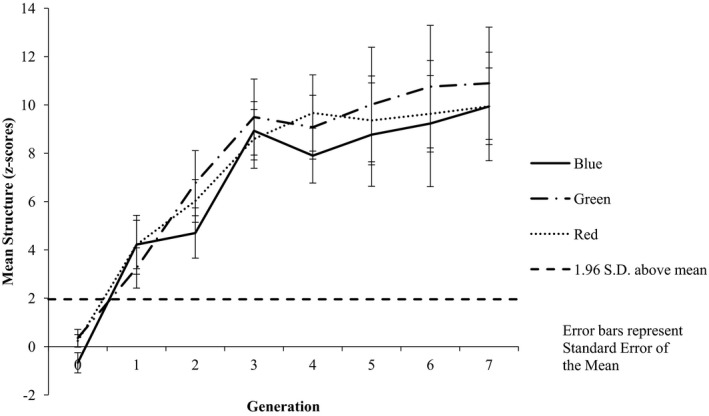
Mean level of structure at each generation by color. Increasing positive *z*‐scores indicate increasing structure, with *z*‐scores greater than +1.96 indicating higher structure than would be expected by chance (α = 0.05).

Inspection of the mean structure associated with the individual features of the color category dimension across the generations suggests no obvious bias toward particular colors (see Fig. 7). An 8 (Generation: G0–G7) × 3 (Shared Feature: blue vs. green vs. red) repeated measures anova revealed a main effect of Generation [*F*(7, 70) = 13.74, *p *<* *.001, $\eta_{p}^{2}$ = 0.579], with pairwise comparisons indicating significantly more structure at G7 (*M* = 8.85) than at G0 (*M* = −0.218; *p* = .001). There was no evidence of a main effect of Shared Feature [*F*(2, 20) = 2.61, *p* = .098, $\eta_{p}^{2}$ = 0.207] nor of a significant Generation by Shared Feature interaction [*F*(14, 140) = 1.48, *p* = .13, $\eta_{p}^{2}$ = 0.129].

#### 3.2.3 Color/Attribute relationship frequencies

As in Experiment 1, to establish whether the dominant structure associated with the color category dimension was driven by bias toward existing semantic associations between individual attributes and individual color features, we examined the frequency with which each attribute was paired with each color feature (see *Supplementary Materials 1*). We used one‐sample *t* tests to compare the measured test phase frequency with which each attribute was associated with each color with the frequency that would be expected by chance. Examination of these comparisons (using the Bonferroni method to adjust the critical *p*‐value to account for the 48 multiple comparisons made within color, thus to achieve significance *p* < .001) revealed that seven attributes appeared with an individual color or colors less frequently than would be expected by chance; no attributes appeared with an individual color more often than would be expected by chance. Of the seven attributes that appeared less often than expected by chance, six attributes appeared less frequently for only one color (*detailed* with blue; *giant* with green; *fancy*,* lovely*, and *sparkly* with red), one attribute appeared less frequently with two colors (*curious* with both blue and green), and one attribute appeared less frequently with all three colors (*amazing*). Five of these seven attributes (i.e., *amazing*,* fancy*,* giant*,* lovely*, and *sparkling*) appeared with an individual color or colors less frequently than would be expected by chance even when the Bonferroni method was used to impose a much more conservative correction based on all 144 comparisons (thus to achieve significance *p *<* *.00034).

The aim of Experiment 2 was to examine whether stereotypes for novel objects would form and evolve around the category dimension of color in a similar manner as they did for novel aliens in Experiment 1; the results support this hypothesis. Replicating the findings of Experiment 1 and those of previous research, there was evidence that as information about novel objects passed down the chains it became increasingly simplified, structured, and easier to learn (Kirby et al., 2008; Martin et al., 2014). Crucially, the development of structure for objects largely mirrored the pattern found for social targets from Experiment 1, with significantly more structure associated with the color category dimension than either of the other two category dimensions. Although there was no difference in the amount of structure associated with the category dimensions of color, shape, and movement by the end of the chains, we suggest that this is because color rapidly reaches near‐ceiling structure relatively early in the chains and then plateaus (around G3), whereas shape and movement continue to gradually accrue structure throughout. Once again, there was no evidence to suggest that the greater structure associated with color was driven by either dominance of individual colors or existing semantic relationships between individual colors and specific attributes.

Unlike previous research examining how individuals learn novel object names, the current findings do not support a dominance of the category dimension of shape relative to color (Davidoff & Ostergaard, 1988; Diesendruck & Bloom, 2003; Goldstone, 1995; Graham & Poulin‐Dubois, 1999; Mash, 2006). Similarly, unlike previous research examining how languages for novel objects evolve through social transmission, the current findings do not support an underspecification of the color category dimension (Kirby et al., 2008); rather, as information passed down our chains, color seemed to take on greater importance as a category defining dimension, at least early on. Given that the stimuli used in the current experiment were almost identical to those used by Kirby et al., it seems likely that the divergent results are a consequence of the nature of the tasks performed by participants. Learning the names of objects may engage different learning processes than learning about the properties of objects. For a language to be useful it must be expressive, with different words used to describe different objects. Learning the properties of objects does not require such expressivity, with objects relatively unconstrained in the potential properties they might share. It seems the way category structure accumulates via cumulative cultural evolution is dependent on the task context in which people encounter information.

What is clear from the results of Experiments 1 and 2 is that the dominance of the category dimension color in the cumulative cultural evolution of novel stereotypes is not peculiar to social targets. It is possible that the structure that accumulates around color is driven by the perceived salience of this category dimension and the features within it. There is evidence to suggest that category salience plays a fundamental role in how people construct categories (Ahn & Medin, 1992) and use them to make subsequent judgments (Dick, Wagner, Stellmacher, & Christ, 2005). Indeed Yee, Ahmed, and Thompson‐Schill (2012) show that the prominence of an object's features can change from one search context to another and argue that an object's conceptual representation is dynamically affected by contextual relevance. Recent research suggests perceived category salience might be pivotal to the development of underspecification in the evolution of artificial languages (Silvey et al., 2015); category dimensions that are manipulated to be less salient are more likely to become underspecified as information passes down transmission chains. Color may be the most salient dimension in Experiments 1 and 2. Experiment 3 examines this possibility by reducing the perceived salience of the color category dimension.

## 4 Experiment 3: Categorizing within colors

The aim of Experiment 3 was to explore whether reducing the perceived perceptual difference between color categories attenuates the bias toward dominance of the color category dimension and thereby influences the development of novel stereotypes. Experiment 3 was identical to Experiment 1, with one exception—the features within the color category dimension were changed from discrete color categories (i.e., blue, green, and red) to different shades within a single‐color category (e.g., lighter blue, mid blue, and darker blue). If people's bias toward categorizing based on color is independent of the perceptual salience of this dimension, we would expect to find the evolution of greater structure associated with color than either of the other two dimensions. However, if people's bias is associated with category dimension salience, we would expect to see the evolution of less structure associated with color than seen in Experiment 1.

### 4.1 Method

#### 4.1.1 Participants

A new sample of 84 undergraduate participants (65 females) took part in Experiment 3.

#### 4.1.2 Materials

**Figure 8:**
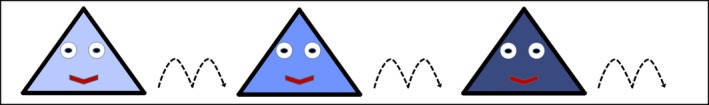
Examples of the aliens in different shades of blue. From left to right; light blue, mid blue, and dark blue.

The materials were identical to those described in the Experiment 1 with the following exception. *Alien Images:* We manipulated the original alien images using Adobe Photoshop to change their color so that they were no longer indicative of discrete color categories (i.e., blue, green, and red) but instead were indicative of shades within a single‐color category (e.g., lighter blue, mid blue, and darker blue). Using the original alien colors as a base (i.e., mid‐color), we used Adobe Photoshop to manipulate the color space values (i.e., Red/Green/Blue: RGB); to create the lighter version of the color, the hue lightness value per pixel of the RGB space was increased by +50; to create the darker version of the color, the hue lightness value per pixel of the RGB space was reduced by −50. The base category color of aliens (blue, red, or green) was counterbalanced across the chains. An example of the different shades of color stimuli is shown in Fig. 8.

#### 4.1.3 Procedure

The procedure was identical to that described in the Experiment 1.

#### 4.1.4 Dependent variables

These were identical to those used in Experiment 1.

### 4.2 Results and discussion

Paired *t* tests revealed that the total number of attributes used to describe all 27 aliens at Generation 7 (*M* = 24) was lower than at Generation 1 (*M* = 39; *t*(11) = 3.41, *p *<* *.006). While there was some correspondence between attributes assigned at Generation 0 and those assigned at Generation 7 (*M* = 12.1% of attributes preserved to Generation 7), one‐sample *t* tests revealed this was not greater than would be expected by chance (chance = 12.4%) even for aliens whose attributes were seen during the training phase by the Generation 1 participant [*M* = 12.9%; *t*(11) = 0.413, *p* = .688]; attributes assigned at Generation 0 but unseen by the participant at Generation 1 were no more likely to appear at the end of the chain than expected by chance [*M* = 11.7%; *t*(11) = −0.780, *p* = .452].

Paired *t* tests also revealed greater accuracy for seen aliens at Generation 7 (*M* = 39%) relative to Generation 1 (*M *=* *16%; *t*(11) = 5.85, *p *<* *.001) and unseen aliens at Generation 7 (*M *=* *35%) relative to Generation 1 (*M *=* *12%; *t*(11) = 6.76, *p *<* *.001). These results therefore replicate the pattern found in Experiments 1 and 2.

#### 4.2.1 Structure

**Figure 9:**
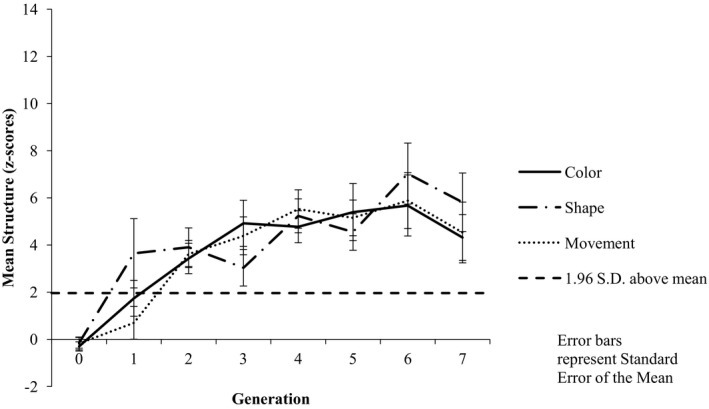
Mean level of structure at each generation by category feature. Increasing positive *z*‐scores indicate increasing structure, with *z*‐scores greater than +1.96 indicating higher structure than would be expected by chance (α = 0.05).

Inspection of the mean structure associated with each category dimension across the generations suggests no bias toward color categorization, with numerically higher structure associated with color in three chains, relative to six chains for shape and three chains for movement (see Fig. 9). The structure data were analyzed using an 8 (Generation: G0–G7) × 3 (Shared Feature: color vs. shape vs. movement) repeated measures anova. The results revealed a main effect of Generation [*F*(7, 77) = 9.63, *p *<* *.001, $\eta_{p}^{2}$ = 0.467], with pairwise comparisons indicating significantly more structure at G7 (*M *=* *4.89) than at G0 (*M *=* *−0.208; *p *<* *.001). There was no evidence of a main effect of Shared Feature [*F*(2, 22)  = 0.77, *p* = .48, $\eta_{p}^{2}$ = 0.065] nor of a Generation by Shared Feature interaction [*F*(14, 154) = 1.18, *p *=* *.30, $\eta_{p}^{2}$ = 0.096].

#### 4.2.2 Color structure comparison between Experiments 3 and 1

Inspection of the mean structure associated with the color category dimension in Experiment 1 with that of Experiment 3 indicates numerically higher structure in Experiment 1 (see Figs. 3 and 4). We compared the color category dimension structure data between Experiments 1 and 3 using an 8 (Generation: G0–G7) × 2 (Experiment 1 vs. 3) mixed factorial anova. The results revealed a main effect of Generation [*F*(7, 154) = 12.71, *p *<* *.001, $\eta_{p}^{2}$ = 0.366]. The results also revealed a main effect of Experiment [*F*(1, 22) = 14.65, *p *=* *.001, $\eta_{p}^{2}$ = 0.400], with significantly more structure associated with color in Experiment 1 (*M* = 6.75) than in Experiment 3 (*M* = 3.77). There was no evidence of a Generation × Experiment interaction [*F*(7, 154) = 1.22, *p *=* *.298, $\eta_{p}^{2}$ = 0.052].

The aim of Experiment 3 was to examine whether, in the current context, the formation of novel stereotypes is driven by an absolute bias toward the category dimension of color or whether it is instead dependent on the relative salience of the different category dimensions. Building on the findings from the first two experiments, the data from Experiment 3 suggest that people's bias to categorize novel targets along a category dimension is modulated by the relative salience of the dimension and that this impacts the way information evolves as it is socially transmitted. Replicating previous findings, there was evidence that as information passed down the chains it became increasingly structured, simplified, and learnable (Experiments 1 and 2; Kirby et al., 2008; Martin et al., 2014). However, unlike in Experiments 1 and 2, there was no difference in the level of structure that accumulated between the different category dimensions. A direct comparison of the structure associated with color in Experiment 3 with the structure associated with color in Experiment 1 indicated significantly less structure had accrued with color in the current experiment. Taken together with the findings of Experiment 2, this suggests that the dominance of the category dimension color found in Experiment 1 is not necessarily a consequence of an absolute bias toward categorizing social targets using color; rather, these findings suggest that people are sensitive to the relative salience of category dimensions when learning information about novel targets and that this sensitivity can have a profound effect on the way information evolves via cumulative cultural evolution.

It is important to note that reducing the salience of the color dimension did not prevent structure accumulating for color—by the end of four of the chains (i.e., a third of the chains we ran) there was numerically higher structure for color than either shape or movement—rather, it reduced the relative dominance exerted by color, as seen in Experiments 1 and 2. This reduction in dominance might be due to an associated reduction in the extent to which individual participants shared a bias toward categorizing the aliens by color. This is consistent with Yee et al. (2012), who suggest that the prominence of features can not only vary across contexts but within a given context, from individual to individual. If fewer people were biased toward categorizing the targets based on color, then one would expect to see less structure developing around the color category dimension across the chains.

When the results from Experiment 3 are considered together with those from Experiment 1, they provide further support for the idea that the relative salience of category dimensions influences the way that information evolves via cumulative cultural evolution (as in Silvey et al., 2015). Just as fewer salient category dimensions are more likely to become underspecified as artificial languages evolve, so relatively fewer salient category dimensions exert smaller influence on the evolution of novel stereotypes. However, the current results also extend previous findings in an important way. Silvey et al. reduced the salience of a category dimension by manipulating the context in which it appeared to ensure that encoding that category dimension in the linguistic signal would be uninformative. The evolving underspecification they observed for the “backgrounded” category dimension was seemingly driven by people's sensitivity to the informativity of encoding the backgrounded dimension; in this way, the backgrounded dimension was conceptually less salient in a meaningful way. Because the salience of the color category dimension was changed between Experiments 1 and 3 without changing the task faced by our participants, the observed differences in the way information evolves are driven by differences in perceptual salience that are unrelated to requirements of the task (i.e., the three shades of colors in Experiment 3 were just as “relevant” as the three discrete colors of Experiment 1; yet they exerted significantly less influence across the chains). It is also possible that the reduction in color dominance might be related to the ease with which people were able to label colors we used. For example, evidence suggests that color terms are organized around universal focal colors and that color language favors percepts from a restricted region of color space (Berlin & Kay, 1969; Regier, Kay, & Cook, 2005). Therefore, although people could perceptually distinguish between the different shades of color, it is probable that fewer people would share the propensity to label differing shades of the same color (e.g., “light blue,” “dark blue”) than would share the propensity to label categorically distinct colors (e.g., “red,” “green”).

## 5 General discussion

Across three studies, we found that as information passed from person to person it became increasingly categorically structured, simplified, and easier to learn until novel category stereotypes had formed. We found that the category dimension of color dominated the formation of novel stereotypes when the experimental context required people to remember information about social targets (Experiment 1), and when the experimental context required people to remember information about objects (Experiment 2). However, there was evidence that the dominance of the color category dimension was diminished when the relative salience of this dimension was perceptually attenuated (Experiment 3). There was no evidence to suggest that the evolving stereotype structure was driven by bottom‐up relationships between the category‐attribute assignments at the start of the chains. Nor was there any evidence that the structure was driven by top‐down knowledge of existing semantic relationships between categories and attributes. When considered alongside previous findings (Beckner et al., 2017; Kirby et al., 2008; Silvey et al., 2015), the current research suggests that the context in which people encounter information and the relative salience of category dimensions influences the cumulative cultural evolution of categorical structure.

The data from all three experiments converge to provide further support for the basic premise that information can evolve to become organized categorically as it is socially transmitted and that this process can occur spontaneously and without any obvious intent. Within seven transmission generations, information that was initially random, complex, and difficult to remember was transformed into an easily learnable system (Kirby et al., 2008; Martin et al., 2014). Faced with the dual problems of an overwhelming amount of information to process and previously unseen targets, participants in our chains made many errors. Crucially, it seems their memory successes and failures were not random; instead, there was some level of categorical structure to their responses. From the beginning of the chains, people overestimated the within‐category similarity of aliens and were more likely to think that aliens who shared features also shared attributes. What began as tiny templates of structure in the episodic recall of one participant were detected and amplified in the recollections of the next participant. Over time these cumulative systematic biases in recall resulted in a coherent categorical structure that could be used efficiently to infer information about targets even when they have never been encountered (Martin et al., 2014).

The data from Experiments 1 and 2 indicate that the color category dimension exerted greater influence than the other two category dimensions on the formation of novel stereotypes for both novel beings and novel objects. By the end of 18 of the 23 transmission chains there was higher structure associated with the color category dimension than with either of the other two dimensions. It seems that people were more likely to overestimate within‐category similarity of targets who shared the same color than they were for targets who shared either the same shape or movement. It is perhaps unsurprising that when the task context required people to remember information about social targets, stereotypes formed around the category dimension of color (Experiment 1). Skin color is a powerful category‐specifying cue of race (e.g., Cloutier et al., 2005), which is one of the most dominant real‐world social category dimensions (Brewer, 1988; Fiske & Neuberg, 1990; Mason et al., 2006). Whether they intended to or not, it is possible that participants in Experiment 1 were more likely to categorize aliens based on color than either movement or shape because color is a highly salient category distinction that is used in everyday life.

However, the results Experiments 2 and 3 suggest that the results in Experiment 1 are unlikely to reflect a specific bias in favor of basing social stereotypes on color, since we see a similar dominance of color for objects in Experiment 2 and a reduced dominance of color in Experiment 3 when targets were still social but color was manipulated to be less salient.

At first glance, the dominance of color stereotypes for objects in Experiment 2 appears inconsistent with research on object categorization, which suggests people are biased toward categorizing objects based on their shape. Because in the real world, shape is more likely to determine object function, people are more likely to assume that novel objects of the same shape (e.g., plates, chairs) are more likely to share functional properties (Davidoff & Ostergaard, 1988; Diesendruck & Bloom, 2003; Goldstone, 1995; Mash, 2006). If people were applying their knowledge of real‐world categorization to novel categorization, one might expect shape to have emerged as the dominant category dimension around which object stereotypes formed. However, the color dominance for object stereotypes found in Experiment 2 might, in part, be due to the fact that of all the attributes used to describe the objects, relatively more are commonly associated with descriptions of color/visual surface (gleaming, polished, shiny) than are commonly associated with descriptions of shape or movement. If the attributes used to describe objects had either conveyed function or had been more commonly associated with shape, then perhaps this category dimension would have proved more salient and would have dominated how novel stereotypes formed.

The results from Experiment 3 demonstrated that novel stereotypes can evolve quite differently when a category dimension is rendered less perceptually salient. By making the color category dimension less salient, we attenuated the apparent dominance this dimension exerted on the formation of novel stereotypes; chains were equally likely to be structured by color, movement, or shape. That is, the three shades of blue used in Experiment 3 are still easy to distinguish, but because they now belong to the same basic color category, or perhaps because they are more perceptually similar to each other, color is less likely to dominate novel stereotype formation. Whether this is a consequence of the colors being more similar to each other and therefore less perceptually salient, or whether this reflects the ease with which people can verbalize category dimensions (e.g., more difficult with dark blue vs. light blue relative to red vs. green) is unknown and requires further investigation. These results support and extend recent evidence that reducing the contextual salience of a category dimension in a novel language learning paradigm leads to underspecification of this dimension (Silvey et al., 2015). Just as Silvey et al. modulated the influence of a category dimension by manipulating its contextual salience, so we modulated the influence of a category dimension by manipulating its perceptual salience. Given the diverse and ever‐changing nature of the human environment, sensitivity to what is currently salient would seem an essential feature for any useful system of categorization. When the results from all three experiments are considered together they provide support for not only for the apparent propensity people share to categorize stimuli in their environment (Fiske & Neuberg, 1990; Rosch, Mervis, Gray, Johnson, & Boyes‐Braem, 1976), but also that the rules that guide this categorization are flexible and sensitive to whatever elements are currently most contextually or perceptually salient (Bernstein, Young, & Hugenberg, 2007; Kowalski & Lo, 2001; Macrae & Bodenhausen, 2000; Silvey et al., 2015).

The way novel stereotypes formed across the three experiments has the potential to inform understanding of real‐world stereotype formation and evolution. For example, dependent on the context and perceptual salience, novel stereotypes were more likely to form around some category dimensions than others. In the real world, some category dimensions appear to exert far greater influence on individual social cognition and consequently society; age, sex, and race are routinely grouped together as being the three most prominent social category dimensions (Brewer, 1988; Fiske, 1993; Fiske & Neuberg, 1990). While there are undoubtedly plausible functional arguments for the pre‐eminence of these category dimensions (e.g., identifying both sex and age are associated with potential reproductive success; Buss, 1989), it might also be the case that these category dimensions tend to dominate much of our stereotypical thinking because they are easily identifiable from faces (Fiske & Neuberg, 1990; Freeman & Ambady, 2011). If the likelihood with which novel stereotypes form around specific category dimensions is dependent on the relative salience of these dimensions, it is quite possible that stereotypes are likely to form and persist for those real‐world category dimensions or individual categories that happen to be most salient, regardless of the functional utility of the emergent stereotypes.

It is also striking that across all three experiments, the content of the novel stereotypes did not form around the attributes that were assigned to targets at the start of the chains. While many aspects of real‐world stereotypes contain a “kernel of truth” (Judd & Park, 1993; Madon et al., 1998), based as they are on a genuine over‐representation of characteristics among members of a social category (e.g., the stereotype of Scottish people includes having red hair, and, relative to people from other nations, Scots are indeed more likely to have red hair), there are other aspects of stereotypes that are of no obvious origin (e.g., the stereotype of Scottish people being miserly). The current results suggest that through a process of cumulative cultural evolution, specific attributes can become strongly associated with category membership without any genuine relationship existing (Martin et al., 2014).

## 6 Conclusion

The experiments reported here support the idea that the formation of novel stereotypes via cumulative cultural evolution is influenced by the context in which people encounter information and the perceptual salience of object features. People are clearly more likely to organize information along some category dimensions than others—in this case color—and this propensity influences the way that novel stereotypes form. However, by modulating the contextual or perceptual salience of different dimensions it was possible to directionally influence how novel stereotypes form. Extrapolating from these results it seems likely that the category structures that pervade human culture reflect the extent to which people share a propensity to categorize their environment in a similar way.

## Supporting information

List of attributes used in Experiment 1.
List of attributes used in Experiment 2.

## Notes

1 Martin et al. (2014) reported collapsed data from two experiments, analyzed for structure and accuracy rather than color bias. The data reported in Experiment 1 are from the first experiment (12 chains of participants). However, analysis of the second experiment included in Martin et al.'s (2014) analysis revealed an identical pattern of results to those reported here.

2 Martin et al. (2014) reported accuracy, the number of attributes used, and category structure by shared feature but did not report analysis of the structure by category dimension. This study extends Martin et al. by including additional analyses to examine whether color dominates novel stereotype formation in the current context and if so whether any one color dominates this process, and examines whether any bias toward color is driven by existing semantic associations between certain colors and individual attributes.
