# Supplementary material for: Context and Perceptual Salience Influence the Formation of Novel Stereotypes via Cumulative Cultural Evolution
Source: Cogn Sci. 2017 Nov 2;42(Suppl Suppl 1):186–212. doi: 10.1111/cogs.12560 (PMC5969227; doi:10.1111/cogs.12560)
Supplement: Supplementary file 1 — List of attributes used in Experiment 1. List of attributes used in Experiment 2. [file COGS-42-186-s001.docx]

**Supplementary Materials S1**

List of attributes used in Experiment 1.

| Adaptable | Confident | Offensive | Sensible |
| --- | --- | --- | --- |
| Adventurous | Curious | Organised | Sensitive |
| Affectionate | Easy-going | Passionate | Serious |
| Aggressive | Excitable | Passive | Shy |
| Ambitious | Flirty | Patient | Successful |
| Anxious | Friendly | Playful | Tactful |
| Arrogant | Hostile | Private | Talented |
| Bitter | Imaginative | Proud | Thoughtless |
| Boring | Jealous | Reliable | Tidy |
| Brave | Lonely | Reserved | Troublesome |
| Bullying | Nasty | Rude | Vulgar |
| Cheerful | nervous | selfish | warm |

List of attributes used in Experiment 2.

| Amazing | Expensive | Heavy | Scented |
| --- | --- | --- | --- |
| Bright | Fancy | Imperfect | Shiny |
| Broad | Firm | Interesting | Solid |
| Clean | Flawed | Large | Sparkly |
| Cold | Flexible | Lovely | Special |
| Colourful | fragrant | Metallic | strong |
| Complex | Genuine | Old | Thick |
| Curious | Giant | Polished | Uneven |
| Dense | Gleaming | Practical | Unique |
| Detailed | Glossy | Rare | Unusual |
| Distinct | Gorgeous | Reflective | Useful |
| Essential | Handy | Robust | Valuable |

|  | **Blue Targets** | | |  | **Green Targets** | | |  | **Red Targets** | | |
| --- | --- | --- | --- | --- | --- | --- | --- | --- | --- | --- | --- |
|  | Mean | SD | *t* |  | Mean | SD | *t* |  | Mean | SD | *t* |
| adaptable | 0.039 | 0.043 | 1.47 |  | 0.034 | 0.040 | 1.21 |  | 0.037 | 0.041 | 1.38 |
| adventurous | 0.037 | 0.035 | 1.66 |  | 0.027 | 0.027 | 0.77 |  | 0.033 | 0.034 | 1.22 |
| affectionate | 0.017 | 0.029 | -0.52 |  | 0.017 | 0.029 | -0.51 |  | 0.025 | 0.031 | 0.44 |
| aggressive | 0.025 | 0.023 | 0.63 |  | 0.018 | 0.018 | -0.55 |  | 0.028 | 0.022 | 1.14 |
| ambitious | 0.025 | 0.015 | 0.91 |  | 0.026 | 0.013 | 1.40 |  | 0.034 | 0.031 | 1.43 |
| anxious | 0.015 | 0.014 | -1.43 |  | 0.024 | 0.017 | 0.67 |  | 0.012 | 0.013 | -2.28* |
| arrogant | 0.023 | 0.027 | 0.30 |  | 0.017 | 0.016 | -0.86 |  | 0.022 | 0.019 | 0.23 |
| bitter | 0.008 | 0.014 | -3.29** |  | 0.007 | 0.012 | -3.97** |  | 0.010 | 0.019 | -2.05 |
| boring | 0.023 | 0.017 | 0.48 |  | 0.041 | 0.038 | 1.88 |  | 0.017 | 0.018 | -0.79 |
| brave | 0.030 | 0.029 | 1.07 |  | 0.016 | 0.015 | -1.14 |  | 0.020 | 0.021 | -0.09 |
| bullying | 0.029 | 0.024 | 1.25 |  | 0.021 | 0.023 | 0.02 |  | 0.039 | 0.042 | 1.56 |
| cheerful | 0.022 | 0.013 | 0.34 |  | 0.021 | 0.014 | 0.09 |  | 0.026 | 0.015 | 1.24 |
| confident | 0.021 | 0.020 | 0.02 |  | 0.017 | 0.012 | -0.97 |  | 0.022 | 0.025 | 0.15 |
| curious | 0.018 | 0.024 | -0.46 |  | 0.016 | 0.021 | -0.85 |  | 0.014 | 0.018 | -1.35 |
| easy-going | 0.008 | 0.010 | -4.47*** |  | 0.009 | 0.008 | -4.99*** |  | 0.009 | 0.009 | -4.51*** |
| excitable | 0.028 | 0.022 | 1.07 |  | 0.026 | 0.021 | 0.96 |  | 0.029 | 0.020 | 1.43 |
| flirty | 0.012 | 0.020 | -1.47 |  | 0.010 | 0.016 | -2.41* |  | 0.028 | 0.039 | 0.60 |
| friendly | 0.026 | 0.025 | 0.73 |  | 0.019 | 0.022 | -0.22 |  | 0.027 | 0.024 | 0.93 |
| hostile | 0.021 | 0.025 | 0.02 |  | 0.021 | 0.031 | 0.07 |  | 0.028 | 0.028 | 0.92 |
| imaginative | 0.013 | 0.013 | -2.00 |  | 0.014 | 0.015 | -1.58 |  | 0.008 | 0.007 | -5.83*** |
| jealous | 0.015 | 0.012 | -1.88 |  | 0.014 | 0.013 | -1.87 |  | 0.015 | 0.013 | -1.59 |
| lonely | 0.030 | 0.021 | 1.52 |  | 0.051 | 0.039 | 2.75* |  | 0.016 | 0.012 | -1.46 |
| nasty | 0.020 | 0.024 | -0.11 |  | 0.014 | 0.018 | -1.25 |  | 0.023 | 0.024 | 0.37 |
| nervous | 0.019 | 0.019 | -0.38 |  | 0.033 | 0.024 | 1.74 |  | 0.015 | 0.015 | -1.32 |
| offensive | 0.009 | 0.011 | -3.90** |  | 0.005 | 0.005 | -10.95*** |  | 0.005 | 0.006 | -9.46*** |
| organised | 0.030 | 0.029 | 1.14 |  | 0.036 | 0.030 | 1.82 |  | 0.032 | 0.026 | 1.51 |
| passionate | 0.018 | 0.020 | -0.48 |  | 0.012 | 0.017 | -1.71 |  | 0.023 | 0.030 | 0.22 |
| passive | 0.019 | 0.022 | -0.22 |  | 0.020 | 0.017 | -0.20 |  | 0.015 | 0.015 | -1.26 |
| patient | 0.007 | 0.006 | -8.13*** |  | 0.010 | 0.009 | -4.35*** |  | 0.005 | 0.003 | -15.88*** |
| playful | 0.024 | 0.021 | 0.57 |  | 0.021 | 0.016 | 0.03 |  | 0.024 | 0.018 | 0.63 |
| private | 0.015 | 0.013 | -1.49 |  | 0.016 | 0.015 | -1.03 |  | 0.010 | 0.010 | -3.53** |
| proud | 0.030 | 0.033 | 0.93 |  | 0.026 | 0.029 | 0.66 |  | 0.020 | 0.018 | -0.14 |
| reliable | 0.029 | 0.025 | 1.11 |  | 0.021 | 0.016 | -0.07 |  | 0.020 | 0.019 | -0.22 |
| reserved | 0.009 | 0.008 | -5.58*** |  | 0.020 | 0.018 | -0.10 |  | 0.011 | 0.007 | -4.88*** |
| rude | 0.030 | 0.026 | 1.18 |  | 0.023 | 0.022 | 0.38 |  | 0.040 | 0.046 | 1.46 |
| selfish | 0.018 | 0.014 | -0.77 |  | 0.021 | 0.013 | 0.04 |  | 0.020 | 0.015 | -0.26 |
| sensible | 0.011 | 0.012 | -2.79* |  | 0.008 | 0.009 | -4.81*** |  | 0.009 | 0.011 | -3.69** |
| sensitive | 0.009 | 0.006 | -6.76*** |  | 0.018 | 0.025 | -0.41 |  | 0.005 | 0.005 | -11.29*** |
| serious | 0.018 | 0.015 | -0.72 |  | 0.015 | 0.015 | -1.29 |  | 0.014 | 0.016 | -1.48 |
| shy | 0.021 | 0.024 | 0.05 |  | 0.042 | 0.040 | 1.84 |  | 0.015 | 0.016 | -1.31 |
| successful | 0.034 | 0.037 | 1.26 |  | 0.027 | 0.028 | 0.72 |  | 0.034 | 0.029 | 1.54 |
| tactful | 0.012 | 0.016 | -1.87 |  | 0.015 | 0.016 | -1.36 |  | 0.014 | 0.013 | -1.77 |
| talented | 0.013 | 0.017 | -1.66 |  | 0.011 | 0.013 | -2.65* |  | 0.013 | 0.017 | -1.57 |
| thoughtless | 0.017 | 0.016 | -0.74 |  | 0.014 | 0.014 | -1.75 |  | 0.010 | 0.014 | -2.57* |
| tidy | 0.030 | 0.034 | 0.89 |  | 0.034 | 0.034 | 1.30 |  | 0.020 | 0.027 | -0.07 |
| troublesome | 0.024 | 0.036 | 0.31 |  | 0.016 | 0.033 | -0.47 |  | 0.018 | 0.024 | -0.36 |
| vulgar | 0.039 | 0.036 | 1.79 |  | 0.045 | 0.047 | 1.78 |  | 0.037 | 0.036 | 1.58 |
| warm | 0.011 | 0.019 | -1.87 |  | 0.010 | 0.017 | -2.09 |  | 0.048 | 0.052 | 1.80 |

***** *p* < .05; ****** *p* < .01; ******* *p* < .001

One sample t-tests results for Experiment 1 comparing the proportionate frequency with which attributes appear at test with each target color relative to the proportionate frequency with which they would be expected to appear by chance (i.e., 0.021). Positive t-test values indicate frequencies that are numerically greater than chance, negative t-test values indicate frequencies that are numerically smaller than chance.

|  | **Blue Targets** | | |  | **Green Targets** | | |  | **Red Targets** | | |
| --- | --- | --- | --- | --- | --- | --- | --- | --- | --- | --- | --- |
|  | Mean | SD | *t* |  | Mean | SD | *t* |  | Mean | SD | *t* |
| amazing | 0.004 | 0.007 | -7.66*** |  | 0.006 | 0.010 | -4.76*** |  | 0.005 | 0.006 | -9.08*** |
| bright | 0.017 | 0.032 | -0.36 |  | 0.024 | 0.043 | 0.24 |  | 0.020 | 0.031 | -0.11 |
| broad | 0.016 | 0.028 | -0.56 |  | 0.011 | 0.020 | -1.61 |  | 0.017 | 0.022 | -0.61 |
| clean | 0.019 | 0.019 | -0.28 |  | 0.036 | 0.040 | 1.29 |  | 0.011 | 0.014 | -2.31* |
| cold | 0.066 | 0.056 | 2.70* |  | 0.063 | 0.057 | 2.43* |  | 0.017 | 0.019 | -0.63 |
| colorful | 0.021 | 0.024 | 0.09 |  | 0.020 | 0.028 | -0.10 |  | 0.033 | 0.042 | 1.00 |
| complex | 0.016 | 0.021 | -0.81 |  | 0.012 | 0.015 | -1.83 |  | 0.016 | 0.018 | -0.96 |
| curious | 0.008 | 0.010 | -4.34*** |  | 0.008 | 0.009 | -4.66*** |  | 0.011 | 0.014 | -2.32* |
| dense | 0.032 | 0.029 | 1.22 |  | 0.024 | 0.027 | 0.43 |  | 0.028 | 0.021 | 1.19 |
| detailed | 0.008 | 0.009 | -4.91*** |  | 0.009 | 0.011 | -3.55** |  | 0.010 | 0.011 | -3.26** |
| distinct | 0.014 | 0.012 | -1.69 |  | 0.013 | 0.012 | -2.32* |  | 0.016 | 0.011 | -1.55 |
| essential | 0.014 | 0.024 | -0.88 |  | 0.014 | 0.020 | -1.18 |  | 0.012 | 0.021 | -1.46 |
| expensive | 0.011 | 0.018 | -1.77 |  | 0.016 | 0.023 | -0.65 |  | 0.011 | 0.011 | -3.06* |
| fancy | 0.012 | 0.017 | -1.73 |  | 0.014 | 0.021 | -1.01 |  | 0.005 | 0.005 | -9.49*** |
| firm | 0.014 | 0.020 | -1.07 |  | 0.016 | 0.017 | -0.86 |  | 0.019 | 0.022 | -0.27 |
| flawed | 0.016 | 0.026 | -0.65 |  | 0.012 | 0.019 | -1.53 |  | 0.013 | 0.022 | -1.24 |
| flexible | 0.014 | 0.016 | -1.34 |  | 0.012 | 0.015 | -2.07 |  | 0.014 | 0.013 | -1.75 |
| fragrant | 0.031 | 0.028 | 1.17 |  | 0.038 | 0.025 | 2.24* |  | 0.030 | 0.032 | 0.96 |
| genuine | 0.020 | 0.022 | -0.12 |  | 0.019 | 0.017 | -0.45 |  | 0.023 | 0.023 | 0.32 |
| giant | 0.012 | 0.014 | -2.06 |  | 0.007 | 0.008 | -5.61*** |  | 0.017 | 0.020 | -0.68 |
| gleaming | 0.018 | 0.020 | -0.53 |  | 0.019 | 0.023 | -0.26 |  | 0.011 | 0.014 | -2.34* |
| glossy | 0.022 | 0.022 | 0.20 |  | 0.030 | 0.026 | 1.17 |  | 0.018 | 0.024 | -0.37 |
| gorgeous | 0.028 | 0.032 | 0.76 |  | 0.032 | 0.034 | 1.04 |  | 0.025 | 0.029 | 0.53 |
| handy | 0.011 | 0.012 | -2.79* |  | 0.010 | 0.012 | -2.83* |  | 0.015 | 0.012 | -1.44 |
| heavy | 0.026 | 0.037 | 0.49 |  | 0.017 | 0.028 | -0.42 |  | 0.035 | 0.039 | 1.24 |
| imperfect | 0.021 | 0.026 | 0.08 |  | 0.018 | 0.020 | -0.41 |  | 0.023 | 0.025 | 0.27 |
| interesting | 0.015 | 0.025 | -0.75 |  | 0.017 | 0.031 | -0.46 |  | 0.017 | 0.024 | -0.54 |
| large | 0.021 | 0.029 | 0.04 |  | 0.021 | 0.028 | 0.07 |  | 0.027 | 0.028 | 0.78 |
| lovely | 0.012 | 0.016 | -1.86 |  | 0.010 | 0.013 | -2.75* |  | 0.008 | 0.007 | -5.77*** |
| metallic | 0.025 | 0.030 | 0.52 |  | 0.023 | 0.020 | 0.38 |  | 0.032 | 0.033 | 1.13 |
| old | 0.026 | 0.031 | 0.58 |  | 0.028 | 0.033 | 0.69 |  | 0.033 | 0.032 | 1.23 |
| polished | 0.023 | 0.019 | 0.35 |  | 0.023 | 0.018 | 0.33 |  | 0.022 | 0.024 | 0.19 |
| practical | 0.018 | 0.021 | -0.47 |  | 0.016 | 0.021 | -0.73 |  | 0.018 | 0.021 | -0.50 |
| rare | 0.036 | 0.023 | 2.16 |  | 0.034 | 0.026 | 1.76 |  | 0.042 | 0.023 | 3.12* |
| reflective | 0.012 | 0.023 | -1.33 |  | 0.013 | 0.021 | -1.26 |  | 0.011 | 0.021 | -1.59 |
| robust | 0.032 | 0.025 | 1.46 |  | 0.029 | 0.025 | 1.09 |  | 0.046 | 0.025 | 3.25** |
| scented | 0.018 | 0.015 | -0.61 |  | 0.031 | 0.033 | 1.00 |  | 0.018 | 0.022 | -0.43 |
| shiny | 0.022 | 0.023 | 0.16 |  | 0.028 | 0.027 | 0.92 |  | 0.015 | 0.015 | -1.18 |
| solid | 0.038 | 0.029 | 1.98 |  | 0.034 | 0.028 | 1.63 |  | 0.046 | 0.030 | 2.73* |
| sparkly | 0.012 | 0.014 | -1.96 |  | 0.013 | 0.018 | -1.37 |  | 0.006 | 0.005 | -9.19*** |
| special | 0.011 | 0.012 | -2.86* |  | 0.012 | 0.014 | -2.14 |  | 0.010 | 0.010 | -3.66** |
| strong | 0.032 | 0.036 | 1.03 |  | 0.026 | 0.033 | 0.52 |  | 0.046 | 0.038 | 2.25* |
| thick | 0.026 | 0.026 | 0.63 |  | 0.017 | 0.019 | -0.65 |  | 0.028 | 0.025 | 0.88 |
| uneven | 0.029 | 0.033 | 0.78 |  | 0.029 | 0.035 | 0.77 |  | 0.023 | 0.028 | 0.25 |
| unique | 0.035 | 0.024 | 1.97 |  | 0.037 | 0.026 | 2.11 |  | 0.034 | 0.028 | 1.54 |
| unusual | 0.023 | 0.019 | 0.39 |  | 0.024 | 0.022 | 0.45 |  | 0.024 | 0.022 | 0.43 |
| useful | 0.023 | 0.030 | 0.22 |  | 0.017 | 0.024 | -0.51 |  | 0.023 | 0.030 | 0.23 |
| valuable | 0.018 | 0.021 | -0.39 |  | 0.017 | 0.021 | -0.55 |  | 0.018 | 0.018 | -0.58 |

***** *p* < .05; ****** *p* < .01; ******* *p* < .001

One sample t-tests results for Experiment 2 comparing the proportionate frequency with which attributes appear at test with each target color relative to the proportionate frequency with which they would be expected to appear by chance (i.e., 0.021). Positive t-test values indicate frequencies that are numerically greater than chance, negative t-test values indicate frequencies that are numerically smaller than chance.
